# Using Bayesian spatial models to map and to identify geographical hotspots of multidrug-resistant tuberculosis in Portugal between 2000 and 2016

**DOI:** 10.1038/s41598-020-73759-w

**Published:** 2020-10-06

**Authors:** Olena Oliveira, Ana Isabel Ribeiro, Elias Teixeira Krainski, Teresa Rito, Raquel Duarte, Margarida Correia-Neves

**Affiliations:** 1 Life and Health Sciences Research Institute (ICVS), School of Medicine, University of Minho, 4710-057 Braga, Portugal; 2 ICVS/3B, PT Government Associate Laboratory, University of Minho, Braga/Guimarães, Portugal; 3 EPIUnit, Instituto de Saúde Pública da Universidade do Porto, Porto, Portugal; 4 Departamento de Ciências da Saúde Pública e Forenses e Educação Médica, Faculdade de Medicina, Universidade do Porto, Porto, Portugal; 5 Department of Statistics, Federal University of Paraná, Curitiba, Paraná Brasil

**Keywords:** Tuberculosis, Epidemiology

## Abstract

Multidrug-resistant tuberculosis (MDR-TB) is a major threat to the eradication of tuberculosis. TB control strategies need to be adapted to the necessities of different countries and adjusted in high-risk areas. In this study, we analysed the spatial distribution of the MDR- and non-MDR-TB cases across municipalities in Continental Portugal between 2000 and 2016. We used Bayesian spatial models to estimate age-standardized notification rates and standardized notification ratios in each area, and to delimitate high- and low-risk areas, those whose standardized notification ratio is significantly above or below the country’s average, respectively. The spatial distribution of MDR- and non-MDR-TB was not homogeneous across the country. Age-standardized notification rates of MDR-TB ranged from 0.08 to 1.20 and of non-MDR-TB ranged from 7.73 to 83.03 notifications per 100,000 population across the municipalities. We identified 36 high-risk areas for non-MDR-TB and 8 high-risk areas for MDR-TB, which were simultaneously high-risk areas for non-MDR-TB. We found a moderate correlation (ρ = 0.653; 95% CI 0.457–0.728) between MDR- and non-MDR-TB standardized notification ratios. We found heterogeneity in the spatial distribution of MDR-TB across municipalities and we identified priority areas for intervention against TB. We recommend including geographical criteria in the application of molecular drug resistance to provide early MDR-TB diagnosis, in high-risk areas.

## Introduction

In Europe, the incidence of tuberculosis (TB) has been decreasing since 2008, at a rate of about 5% per year. In 2017 the incidence of TB was 30 new cases per 100,000 population. Although this rate of decline was higher than the global rate of decline of incidence (currently at 2%), it still needs to be improved to achieve the goals of the End TB Strategy^1^.

Multidrug-resistant tuberculosis (MDR-TB) is defined as TB caused by strains of *Mycobacterium tuberculosis* resistant to the two most potent first-line anti-TB drugs, isoniazid and rifampicin. It contributes to the difficulty in achieving the goals of the End TB Strategy. MDR-TB treatment requires the use of second-line anti-TB drugs, which are less effective, more toxic and more costly^2^, with a lower success rates than standard therapy^1^. Hence, prevention and control of MDR-TB are priorities for elimination of TB^3^.

Since the 1990’s, when MDR-TB was recognised as a potential threat to TB control, it was considered that in general, drug-resistance was acquired during treatment due to poor quality case management^4^. Currently, the premise that drug-resistant TB is predominantly acquired has changed. The use of new epidemiological tools, such as modelling and molecular techniques, demonstrates that the majority of MDR-TB cases result from transmission of MDR-TB strains rather than selection of de-novo resistance during previous treatment^5,6^.

In 2017, in Europe, MDR-TB was reported for 24% of all TB cases with first-line drug susceptibility testing (DST). This proportion was considerably lower among European Union (EU) countries (4%) compared with non-EU countries (28%). Among pulmonary TB cases, 18% of new and 48% of previously treated cases were MDR-TB^1^. In the same year, in Portugal, the incidence of TB was 16 cases per 100,000 population and 1% of all TB cases were MDR-TB. However, TB incidence was not homogeneous across the country, with 57% of cases in the two largest urban centers, Porto and Lisbon^7^. These cities were previously identified as the most critical regions for TB incidence^8^, and pulmonary TB in particular^9,10^. Adaptation of strategies and interventions to national and local contexts is pivotal for effective TB control^11^. This can only be achieved with a detailed understanding of the disease distribution across the different regions, along with an epidemiological characterization of the populations affected, paying special attention to the identification of geographical areas or subpopulations with especially high TB burden^11^. Spatial statistics and disease mapping are effective approaches to investigate the detailed geographical variations in TB incidence^12^, being particularly relevant in identifying high- and low-risk areas^8,13^.

In the present study, we analysed the spatial distribution of notification of TB in municipalities in Continental Portugal to identify high-risk areas for MDR- and non-MDR-TB. We also assessed the correlation between the spatial distributions of MDR- and non-MDR-TB, highlighting populations that could be major targets for public health authorities to reduce and prevent the incidence of MDR-TB in Portugal.

## Methods

### Data collection

We used the national TB Surveillance System (SVIG-TB) as the source of data. We analysed all TB cases notified in Continental Portugal from January 2000 until December 2016. According to national regulations, 2 independent sputum samples are collected and tested. TB diagnosis is done either through positive identification using microscopy and nucleic acid amplification or positive *Mycobacterium tuberculosis* (Mtb) culture, followed by conventional first-line DST. All tests are performed in laboratories integrated in the national network, periodically certified and checked. All Mtb strains that have shown resistance to isoniazid and rifampicin at the same time should be tested for second-line anti-TB drugs in the TB National Reference Laboratory (Instituto Nacional de Saúde Ricardo Jorge: INSA). In the case of suspicion of MDR-TB (patients with previous TB treatment that report contact with MDR-TB patients, that belong to specific vulnerable populations, or that are health professionals), clinical samples are submitted to molecular testing for detection of isoniazid and rifampicin resistance.

We selected MDR-TB cases (i.e., resistant to at least isoniazid and rifampicin) and divided all TB cases into two groups: MDR- and non-MDR-TB cases. We obtained notifications of MDR- and non-MDR-TB by municipality (n = 278), year of diagnosis, age (5-year age groups) and sex. Population counts by municipality, year, age (5-year age groups) and sex were obtained from Statistics Portugal (https://www.ine.pt/) for the study period.

Demographic and clinical characteristics of each patient, including age, sex, country of origin, health-related behaviours (e.g. drug or alcohol abuse), HIV status, reclusion (prison confinement), community residence (social housing for people with socio-economic vulnerabilities), homelessness, comorbidity (diabetes and silicosis), previous TB treatment and site of disease, were also collected from SVIG-TB.

### Statistical analysis

Descriptive statistics [absolute and relative frequencies or median with interquartile range (IQR)], according to the nature of the variables, were used to describe patient characteristics. We compared these characteristics between patient groups using the Chi-squared test (or Fisher’s test, if appropriate) for categorical variables and the Mann–Whitney U-test for continuous variables. In order to control for an effect of the different sample sizes of both groups (MDR and non-MDR, we selected two random samples with 583 cases of the non-MDR to compare with our MDR group).

To estimate age-standardized notification rates in each area and to delimitate high risk and low risk areas, we used hierarchical Bayesian spatial models. These models take into account the spatial autocorrelation and large variance of small areas. To minimize the effect of random fluctuations associated with small number of cases, and because we found no substantial differences in the geographical distribution of non-MDR and MDR TB across our study period, we considered the average rates of the 17-year study period. We assumed that the response variable, cases of TB $({O}_{i})$ in each $i$th area, follows a Poisson distribution where ${E}_{i}$ is the expected number of cases and ${\theta }_{i}$ the relative risk (RR), or Standardized Notification Rate (Eqs. 1 and 2). We used the Portuguese TB notification rates by sex and age group (5-year age groups) as a reference to compute the expected number of cases, according to the indirect method of standardization. The expected number of cases was obtained by summing the product of the age-sex specific notification rates of the standard population (in our study Portugal) by the population by age and sex of each Portuguese municipality.

1 $$O_{i} \sim Poisson \left( {E_{i} , \theta_{i} } \right),$$

2 $$log\left( {\theta_{i} } \right) = \alpha + s_{i} .$$

Here $\alpha$ is an intercept quantifying the average number of TB cases in the 278 areas. The area specific effect $s_{i}$ was modelled considering a BYM model^14^ with a parameterization suggested by Dean et al.^15^ (Eq. 3)

3 $$s_{i} = \tau \left( {\sqrt \varphi \times u_{i} + \sqrt {1 - \varphi } \times v_{i} } \right) ,$$

where $u_{i}$ is the structured effect and $v_{i}$ is the unstructured effect. The $u_{i}$ effect was scaled in order to render the model more intuitive and interpretable^16^, so that $\varphi$ expresses the proportion of the spatial effect due to the structured part and 1/$\tau$ is the marginal variance of $s_{i}$.

Additionally, we used the function ‘excursions’ to delimitate high risk and low risk areas^8,17,18^. High-risk areas are those whose standardized notification ratio is significantly above 1 (i.e., above the country’s average) and low risk areas are those whose standardized notification ratio is significantly below 1 (i.e., below the country’s average). This method uses the posterior joint distribution computed from the Integrated Nested Laplace Approximation (INLA) and takes into account the dependence structure, allowing to accurately identify areas where the notification ratio is greater than zero.

To analyse the correlation between MDR-TB and non-MDR-TB, the Pearson correlation coefficient (r and corresponding 95% Credible Intervals, 95% CrI) was computed based on the standardized notification ratios of MDR-TB and non-MDR-TB derived from the previously described models.

To facilitate interpretation, standardized notification ratios were converted into rates per 100,000 inhabitants by multiplying the standardized notification ratios by the crude national notification rates.

Statistical analyses were performed using SPSS version 18.0 (PASW Statistics 18), and p-values below 0.05 were considered statistically significant.

Posterior distributions were obtained using the INLA, which was implemented in the R INLA library^19^.

Standardized notification rates and high and low risk areas were mapped using ArcMap release 10.5.1. (Environmental Systems Research Institute, Redlands, CA, USA).

### Ethical considerations

Ethical approval and informed consent were not required, as the patient data, collected for an official national surveillance system, were anonymized in accordance with the research ethical guidelines in Portugal. Authorization for its use in the present manuscript was given by the National program for Tuberculosis.

## Results

We evaluated 53,417 TB cases, notified in Continental Portugal during the study period (2000–2016) (Supplementary Table S1). We identified 583 (1.1%) cases of MDR-TB. We compared demographic and clinical characteristics between MDR- and non-MDR-TB patients. We observed that MDR-TB patients were younger (40.0 years vs. 42.0 years) and were more likely to be foreign-born (27.3% vs. 13.6%), infected with HIV (27.8% vs. 13.1%), alcohol abusers (24.5% vs. 15.0%), injectable drug users (20.3% vs. 10.3%), prisoners (6.2% vs. 2.3%), homeless (3.7% vs. 1.8%) and having a history of previous TB treatment (40.3% vs. 9.9%) than non-MDR-TB patients (Table 1). The same statistical differences were obtained with two randomized samples I and II of the non-MDR-TB with similar size as the MDR-TB group (Supplementary Table S2).

**Table 1:** Characteristics of multidrug-resistant tuberculosis (MDR-TB) and non-MDR-TB patients, from cases in Continental Portugal, for the years of 2000–2016.

Patient’s characteristics	Total n	MDR-TB		Non-MDR-TB		*p*-value
		n^a^	IQR or %	n^a^	IQR or %	
**Age (years)**						
Median (IQR)	53,417	40.0	19	42.0	26	0.002^b^
**Gender**						
Female	53,417	174	29.8	17,282	32.7	0.155
Male		409	70.7	35,552	67.3	
**Country of origin**						
Foreign-born	53338^e^	159	27.3	7184	13.7	< 0.001
Native		424	72.2	45,571	86.3	
**HIV status**						
Negative	53,417	421	72.2	45,929	86.9	< 0.001
Positive		162	27.8	6905	13.1	
**Alcohol abuse**^f^						
No	48775^e^	386	75.5	41,045	85.0	< 0.001
Yes		125	24.5	7219	15.0	
**Injectable drug use**^f^						
No	49337^e^	415	79.7	43,780	89.7	< 0.001
Yes		106	20.3	5036	10.3	
**Reclusion**^c^						
No	49482^c^	481	93.8	47,836	97.7	< 0.001
Yes		32	6.2	1133	2.3	
**Community residence**^d^						
No Yes	49271^e^	480	94.6	47,011	96.4	0.084
		26	5.1	1754	3.6	
**Homelessness**						
No Yes	49436^e^	490	96.3	48,058	98.2	0.002
		19	3.7	869	1.8	
**Diabetes**						
No Yes	53,417	557	95.5	50,128	94.9	0.531
		26	4.5	2706	5.1	
**Silicosis**						
No Yes	53,417	578	99.1	52,414	99.2	0.812
		5	0.9	420	0.8	
**Previous TB treatment**						
No	53,417	348	59.7	47,615	90.1	< 0.001
Yes		235	40.3	5219	9.9	
**Site of disease**						
Pulmonary	53263^e^	530	91.1	38,484	73.1	< 0.001
Extra-pulmonary		52	8.9	14,197	26.9	

*TB* tuberculosis, *MDR-TB* multidrug-resistant tuberculosis, *IQR* interquartile range, *HIV* human immunodeficiency virus.

^a^Not applicable for age.

^b^Mann–Whitney U-test.

^c^Prison confinement.

^d^Social housing for people with socio-economic vulnerabilities.

^e^Data missing for: country of origin (n = 79; 0.1%), alcohol abuse (n = 4642; 8.7%), injectable drug use (n = 4080; 7.6%), reclusion (n = 3935; 7.4%), community residence (n = 4146; 7.8%), homelessness (n = 3981; 7.5%), site of disease (n = 154; 0.3%).

^f^Self-reported.

The crude non-MDR-TB notification rate was 31.19 notifications per 100,000 population (95% CrI 30.93–31.46) and the crude MDR-TB notification rate was 0.34 notifications per 100,000 population (95% CrI 0.32–0.37). Geographical differences in reporting were observed.

**Figure 1:**
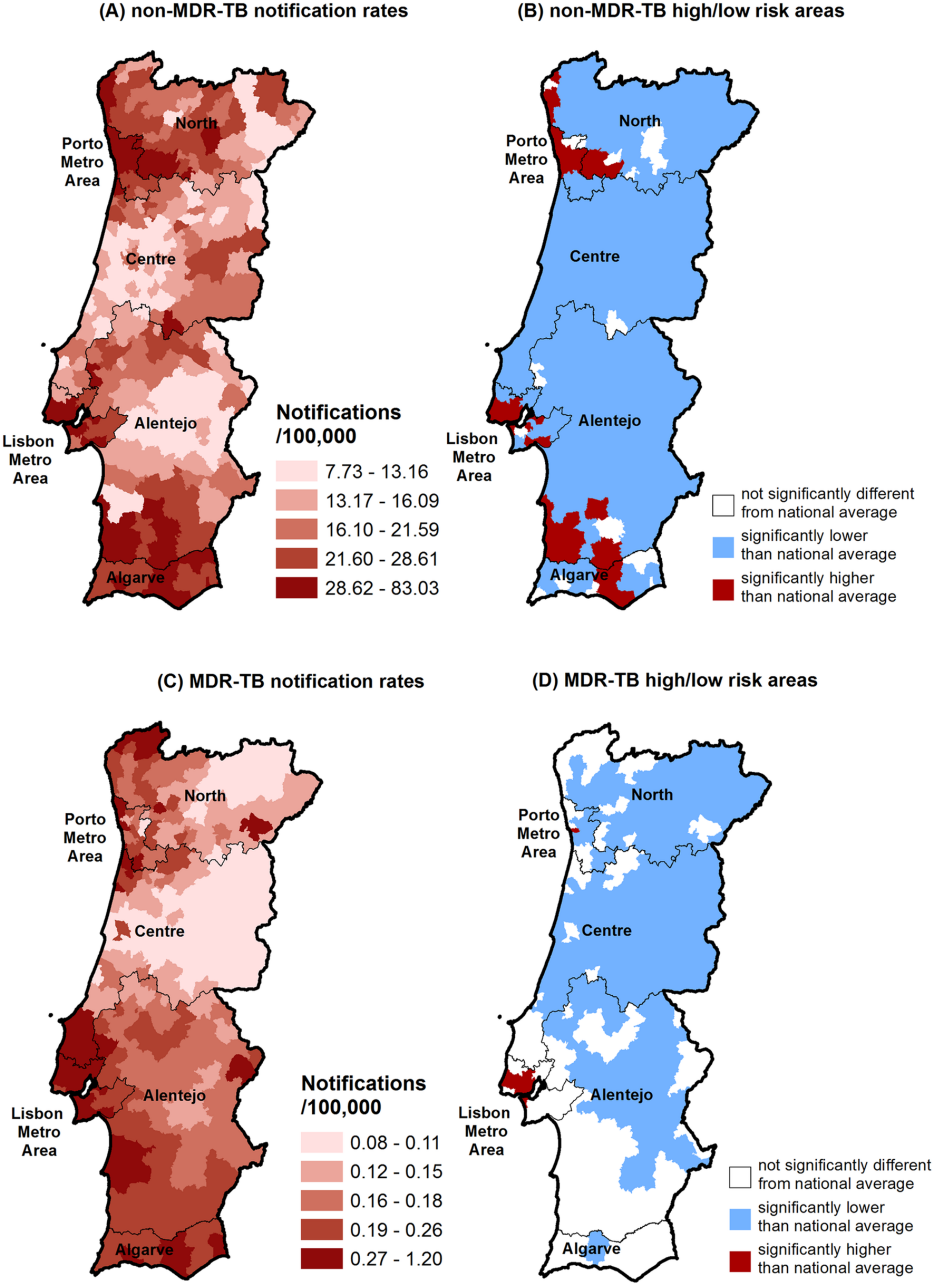
Spatial distribution of the age-standardized notification rates of non-MDR-TB (**A**) and the corresponding delimitation of the high- and low-risk areas (**B**). Spatial distribution of the age-standardized notification rates of MDR-TB (**C**) and the corresponding delimitation of the high- and low-risk areas (**D**). *MDR-TB* multidrug-resistant tuberculosis; high-risk areas are those whose standardized notification ratio is significantly above 1 (i.e., above the country’s average); low risk areas are those whose standardized notification ratio is significantly below 1 (i.e., below the country’s average).

The spatial distribution of the age-standardised notification rates of non-MDR-TB is depicted in Fig. 1A with the delimitation of the high- and low-risk areas given in Fig. 1B. Age-standardized notification rates of non-MDR TB ranged from 7.73 to 83.03 notifications per 100,000 population. We identified 36 high-risk areas, mostly located in Porto and Lisbon metropolitan areas, and also in the southern regions of Alentejo and the Algarve (Fig. 1B). The spatial distribution of the age-standardized notification rates of MDR-TB is shown in Fig. 1C and the delimitation of the high- and low-risk areas is shown in Fig. 1D. Age-standardized notification rates ranged from 0.08 to 1.20 notifications per 100,000 population. Eight high-risk areas for MDR-TB were located mostly in the Lisbon metropolitan area (Fig. 1D). These 8 high-risk areas were also high-risk areas for non-MDR-TB. Only 22% (8/36) of the high-risk areas for non-MDR-TB were high-risk areas for MDR-TB (Supplementary Table S3). In order to confirm the stability of the inferred high-risk areas through the entire dataset, we performed the analysis on a time series across the 17 years. We obtained stable patterns for the geographical locations of risk areas (Supplementary Figs. S1 and S2).

We analysed the correlation between MDR- and non-MDR-TB standardized notification ratios and found a moderate correlation (ρ = 0.653; 95% CrI 0.457–0.728) between them.

Since only some areas with a high-risk for non-MDR-TB also have a high-risk for MDR-TB (Supplementary Table S3), we compared demographic and clinical characteristics of the non-MDR-TB patients from high-risk areas for only non-MDR-TB (28 areas) with patients from areas, which are also high-risk areas for MDR-TB (8 areas) to determine factors that could be associated with the risk for MDR-TB. We observed that the patients from high-risk areas for both MDR- and non-MDR-TB were younger (40.0 years vs. 42.0 years) than patients from areas with highest-risk for only non-MDR-TB. Among them, there was a higher proportion of females (34.6% vs. 31.3%), foreign-born patients (25.5% vs. 7.2%), HIV infection (20.9% vs. 12.2%), alcohol abusers (17.4% vs. 13.9%), injectable drugs users (16.3% vs. 10.2%), prisoners (4.0% vs. 1.5%), community residents (4.9% vs. 2.4%), homeless persons (3.3% vs. 1.1%), cases of extra-pulmonary disease (28.3% vs. 24.5%) and cases with a history of previous TB treatment (12.6% vs. 10.0%) (Table 2).

**Table 2:** Comparison of the characteristics of TB patients between those in high-risk areas only for non-MDR-TB and those in high–risk areas for both MDR- and non-MDR-TB, Continental Portugal, 2000–2016.

Patient’s characteristics	Total n	High-risk areas for non-MDR-TB but not for MDR-TB		High-risk areas for both MDR- and non-MDR-TB		*p*-value
		n^a^	IQR or %	n^a^	IQR or %	
**Age (years)**						
Median (IQR)	32,114	42.0	25	40.0	24	< 0.001^b^
**Gender**						
Female	32,114	4816	31.3	5793	34.6	< 0.001
Male		10,562	68.7	10,943	65.4	
**Country of origin**						
Foreign-born	32,077	1107	7.2	4266	25.5	< 0.001
Native		14,255	92.8	12,449	74.5	
**HIV status**						
Negative	32,114	13,508	87.8	13,240	79.1	< 0.001
Positive		1870	12.2	3496	20.9	
**Alcohol abuse**^f^						
No	28970^e^	11,867	86.1	12,544	82.6	< 0.001
Yes		1911	13.9	2648	17.4	
**Injectable drug use**^f^						
No	29480^e^	12,655	89.8	12,891	83.7	< 0.001
Yes		1431	10.2	2503	16.3	
**Reclusion**^c^						
No	29152^e^	13,895	98.5	14,440	96.0	< 0.001
Yes		217	1.5	600	4.0	
**Community residence**^d^						
No	29005^e^	13,687	97.6	14,259	95.1	< 0.001
Yes		331	2.4	728	4.9	
**Homelessness**						
No	29136^e^	13,940	98.9	14,549	96.7	< 0.001
Yes		148	1.1	499	3.3	
**Diabetes**						
No	32,114	14,654	95.3	15,892	95.0	0.172
Yes		724	4.7	844	5.0	
**Silicosis**						
No	32,114	15,161	98.6	16,720	99.9	< 0.001
Yes		217	1.4	16	0.1	
**Previous TB treatment**						
No	32,114	13,844	90.0	14,631	87.4	< 0.001
Yes		1534	10.0	2105	12.6	
**Site of disease**						
Pulmonary	32011^e^	11,574	75.5	11,961	71.7	< 0.001
Extra-pulmonary		3752	24.5	4724	28.3	

*TB* tuberculosis, *MDR-TB* multidrug-resistant tuberculosis, *IQR* interquartile range, *HIV* human immunodeficiency virus.

^a^Not applicable for age.

^b^Mann–Whitney U-test.

^c^Prison confinement.

^d^Social housing for people with socio-economic vulnerabilities.

^e^Data missing for: country of origin (n = 37; 0.1%), alcohol abuse (n = 3144; 9.8%), injectable drug use (n = 2634; 8.2%), reclusion (n = 2962; 9.2%), community residence (n = 3109; 9.7%), homelessness (n = 2978; 9.3%), site of disease (n = 103; 0.3%).

^f^Self-reported.

## Discussion

In this study, we combined the epidemiological characteristics of MDR- (resistant to at least isoniazid and rifampicin) and non-MDR-TB (all other TB) patients, over a 17-year period, with a detailed spatial description to identify high- and low-risk areas, to obtain a systematic comparison between MDR- and non-MDR-TB high-risk areas across Portugal.

We demonstrated significant heterogeneity in the spatial distribution of the age-standardized notification rates of MDR- and non-MDR-TB at the municipality level. We found a moderate correlation between MDR- and non-MDR-TB standardized notification ratios. We identified 36 high-risk areas for non-MDR-TB and 8 high-risk areas for MDR-TB.

In our study period (2000–2016), the spatial distribution of the age-standardised notification rates of non-MDR-TB ranged from 7.73 to 83.03 notifications per 100,000 population. A high degree of heterogeneity in spatial TB distribution was expected as previously reported in national^8–10^ and international^12^ spatial studies. The spatial distribution of the age-standardised notification rates of MDR-TB was also heterogeneous (up to fifteen times difference), ranging from 0.08 to 1.20 notifications per 100,000 population across municipalities.

The pronounced spatial heterogeneity of MDR-TB burden has been observed in Moldova (the notified incidence of MDR-TB ranged from 0.5 to 27.2 cases per 100,000 population)^20^, China (where the proportion of incident MDR-TB cases varied between 3 and 30%)^21^ and Ethiopia (where the standardized morbidity ratio ranged from 0 to 7.0)^22^.

We found a moderate correlation between MDR- and non-MDR-TB. We identified 36 high-risk areas for non-MDR-TB and 8 high-risk areas for MDR-TB, which were simultaneously high-risk areas for non-MDR-TB. It was expected that MDR-TB risk areas were comparable with non-MDR-TB risk areas, due to the high probability of acquisition of drug resistance during treatment for TB and the transmission of existing MDR-TB strains in areas with higher rate of transmission of non-MDR-TB. However, only 22% (8/36) of the non-MDR-TB high-risk areas were also MDR-TB high-risk areas. We compared non-MDR-TB patients from high-risk areas for non-MDR-TB with patients from high-risk areas for both MDR- and non-MDR-TB. Among the characteristics which were most common among the patients from high-risk areas for both MDR- and non-MDR-TB, being HIV infected^23–26^, being foreign-born^6,23^, homelessness^27^ and having history of imprisonment^6^, consumption of alcohol^6,25^ and injectable drug use^6^ have been previously reported as factors associated with MDR-TB development. Previous TB treatment is particularly important risk factor for MDR-TB^23–27^.

The role of HIV infection as risk factor for MDR-TB has been inconsistent. In several studies, an association between HIV and MDR-TB disease was not significant or was negative^27,28^. This association was stronger for transmitted than acquired MDR-TB^29,30^.

Regarding previous TB treatment, in our study, 40% of MDR-TB patients were previously exposed to anti-tuberculosis drugs. These cannot be assumed to have acquired resistance during treatment. As previously described, 61% of the incidence of MDR-TB among previously treated patients resulted from MDR-TB transmissio^5^. In fact, genetic studies^31–33^ suggested that a high percentage of these cases in Portugal were related with the transmission of two stable MDR-TB clusters.

Regarding hotspots of MDR-TB, 7 out of the 8 high-risk areas are located in the Lisbon metropolitan area. Previously identified MDR-TB genetic clusters revealed evidence of transmission of multidrug-resistant strains in this region^31–33^.

The strengths of this study are the robust statistical methods used to characterise geographic patterns, taking advantage of the epidemiological characterization of the population over a significant amount of time. This allowed the identification of risk areas for MDR-TB, which are areas for priority action and intervention for the existing national TB control program. We complemented the spatial analysis with quality-assured laboratory data and a detailed epidemiological characterization to evaluate potential risk factors for MDR-TB in the TB risk areas. One possible study limitation is its retrospective design using the national notification system, which limited us in the analysis of the study variables.

In conclusion, we found heterogeneity in the spatial distribution of MDR-TB across municipalities in Portugal. We identified priority areas for intervention against MDR-TB. Our findings suggest that in addition to the development of MDR-TB, transmission of MDR-TB strains occurs in these areas. We propose the inclusion of geographical criteria in the application of molecular drug resistance testing, paying particular attention to screening and early MDR-TB diagnosis in these areas and the performance of routine genotyping of all TB isolates to understand the dynamics of MDR-TB emergence and transmission.

## Supplementary information

Supplementary information.

## Data Availability

The epidemiological and geographical datasets generated during the current study are available from the corresponding author on reasonable request.
