# Supplementary material for: Using Bayesian spatial models to map and to identify geographical hotspots of multidrug-resistant tuberculosis in Portugal between 2000 and 2016
Source: Sci Rep. 2020 Oct 6;10:16646. doi: 10.1038/s41598-020-73759-w (PMC7538940; doi:10.1038/s41598-020-73759-w)
Supplement: Supplementary file 1 — Supplementary information. [file 41598_2020_73759_MOESM1_ESM.pdf]

Using Bayesian spatial models to map and to identify geographical hotspots of multidrug-resistant tuberculosis in Portugal between 2000 and 2016

Olena Oliveira, Ana Isabel Ribeiro, Elias Teixeira Krainski, Teresa Rito, Raquel Duarte, Margarida Correia-Neves

Supplementary Table S1. The absolute annual number of multidrug-resistant tuberculosis (MDR-TB) and non-MDR-TB cases, in Continental Portugal, from 2000 to 2016.

Year	MDR-TB	Non-MDR-TB	Total
**2000**	50	4417	4467
**2001**	43	4315	4358
**2002**	47	4528	4575
**2003**	43	4159	4202
**2004**	56	3804	3860
**2005**	45	3536	3581
**2006**	31	3390	3421
**2007**	47	3078	3125
**2008**	30	2851	2881
**2009**	26	2743	2769
**2010**	28	2622	2650
**2011**	33	2499	2532
**2012**	23	2510	2533
**2013**	16	2312	2328
**2014**	25	2160	2185
**2015**	22	2076	2098
**2016**	18	1834	1852
**Total**	**583**	**52834**	**53417**

Supplementary Table S2. Comparison of the patient's characteristics between groups: MDR-TB (583 cases) with non-MDR-TB random sample I (583 cases) and MDR-TB (583 cases) with non-MDR-TB random sample II (583 cases).

**Patient's characteristics**		**MDR-TB** n (IQR or %)	**Non-MDR-TB random sample I** n (IQR or %)	***p-value***	**Non-MDR-TB random sample II** n (IQR or %)	***p-value***
Age (years)	Median (IQR)	40.0 (19)	43.0 (26)	0.015	43.0 (26)	0.004
Gender	Female	174 (29.8)	199 (34.1)	0.132	198 (34.0)	0.148
	Male	409 (70.2)	384 (65.9)		385 (66.0)	
Country of origin	Foreign-born	159 (27.3)	85 (14.6)	<0.001	79 (13.6)	<0.001
	Native	424 (72.7)	498 (85.4)		504 (86.4)	
HIV status	Negative	421 (72.2)	522 (89.5)	<0.001	514 (88.2)	<0.001
	Positive	162 (27.8)	61 (10.5)		69 (11.8)	
Alcohol abuse	No	386 (75.5)	436 (82.1)	0. 012	457 (86.1)	<0.001
	Yes	125 (24.5)	95 (17.9)		74 (13.9)	
Injectable drug use	No	415 (79.7)	481 (89.6)	<0.001	492 (91.1)	<0.001
	Yes	106 (20.3)	56 (10.4)		48 (8.9)	
Reclusion	No	481 (93.8)	523 (98.1)	0.001	529 (98.1)	0.001
	Yes	32 (6.2)	10 (1.9)		10 (1.9)	
Community residence	No	480 (94.6)	514 (96.6)	0.212	523 (96.7)	0.192
	Yes	26 (5.1)	18 (3.4)		18 (3.3)	
Homelessness	No	490 (96.3)	524 (98.3)	0.065	532 (98.0)	0.140
	Yes	19 (3.7)	9 (1.7)		11 (2.0)	
Diabetes	No	557 (95.5)	556 (95.4)	1.000	551 (94.5)	0.501
	Yes	26 (4.5)	27 (4.6)		32 (5.5)	
Silicose	No	578 (99.1)	578 (99.1)	1.000	578 (99.1)	1.000
	Yes	5 (0.9)	5 (0.9)		5 (0.9)	
Previous TB treatment	No	348 (59.7)	515 (88.3)	<0.001	529 (90.7)	<0.001
	Yes	235 (40.3)	68 (11.7)		54 (9.3)	
Site of disease	Pulmonary	530 (91.1)	422 (72.5)	<0.001	415 (71.3)	<0.001
	Extra-pulmonary	52 (8.9)	160 (27.5)		167 (28.7)	

TB=tuberculosis; MDR-TB= multidrug- resistant tuberculosis; n= number of cases; IQR= interquartile range; HIV =human immunodeficiency virus.

Supplementary Table S3. High-risk areas (standardized notification ratio is significantly above 1, i.e., above the country's average) for non-MDR-TB and MDR-TB, Continental Portugal, 2000-2016.

Region	Municipality	Non-MDR-TB (snr)	MDR-TB (snr)
NORTH	Vila Nova de Cerveira	1.21	nd
	Viana do Castelo	1.10	nd
	Esposende	1.32	nd
	Póvoa de Varzim[a]	1.67	nd
	Vila do Conde[a]	1.84	nd
	Gondomar[a]	1.57	nd
	Vila Nova de Gaia[a]	1.20	nd
	Maia[a]	1.27	nd
	Matosinhos[a]	1.64	nd
	Paredes[a]	1.25	nd
	Penafiel	2.66	nd
	Porto[a,b]	2.24	1.71
	Valongo[a]	1.61	nd
	Marco de Canaveses	1.98	nd
	Espinho[a]	1.24	nd
	Castelo de Paiva	1.14	nd
	Cinfães	1.39	nd
	Resende	1.69	nd
LISBON Metropolitan Area	Cascais	1.20	nd
	Lisboa[b]	1.81	3.49
	Loures[b]	1.25	2.12
	Oeiras[b]	1.32	1.90
	Sintra[b]	1.18	2.50
	Amadora[b]	1.89	2.99
	Odivelas[b]	1.24	2.97
	Almada[b]	1.42	1.79
	Alcochete	1.26	nd
	Moita	1.28	nd
	Setúbal	1.25	nd
ALENTEJO	Sines	1.23	nd
	Aljustrel	1.38	nd
	Almodôvar	1.36	nd
	Odemira	1.08	nd
ALGARVE	Faro	1.18	nd
	Loulé	1.37	nd
	Olhão	1.06	nd

TB=tuberculosis; MDR-TB= multidrug- resistant tuberculosis
snr: standardized notification ratio; nd: not significantly different from country's average.
[a] Porto Metropolitan Area.
[b] High-risk areas for both MDR- and non-MDR-TB.

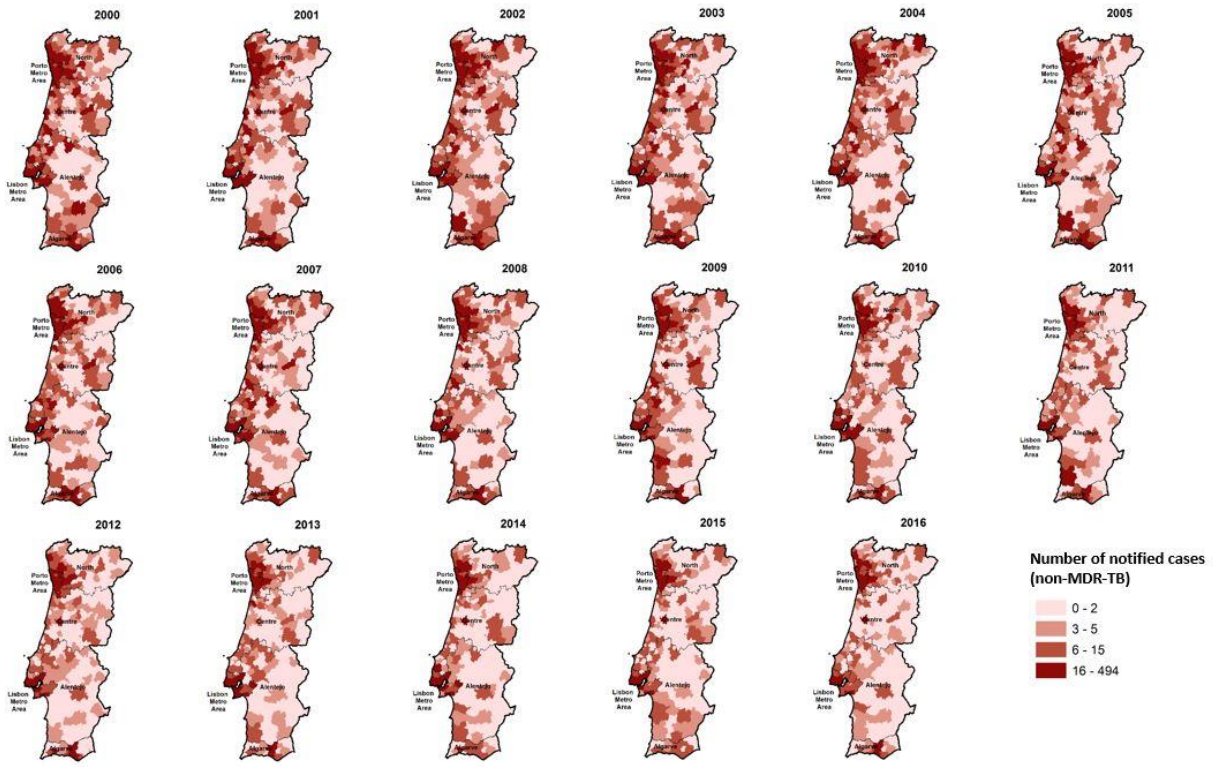

Supplementary Figure S1. Maps depicting a time series of the number of non-MDR-TB cases by municipality year by year, Continental Portugal, 2000-2016. TB=tuberculosis; MDR-TB= multidrug- resistant tuberculosis; the colour code displays the number of notified cases, from 0 to 494 cases.

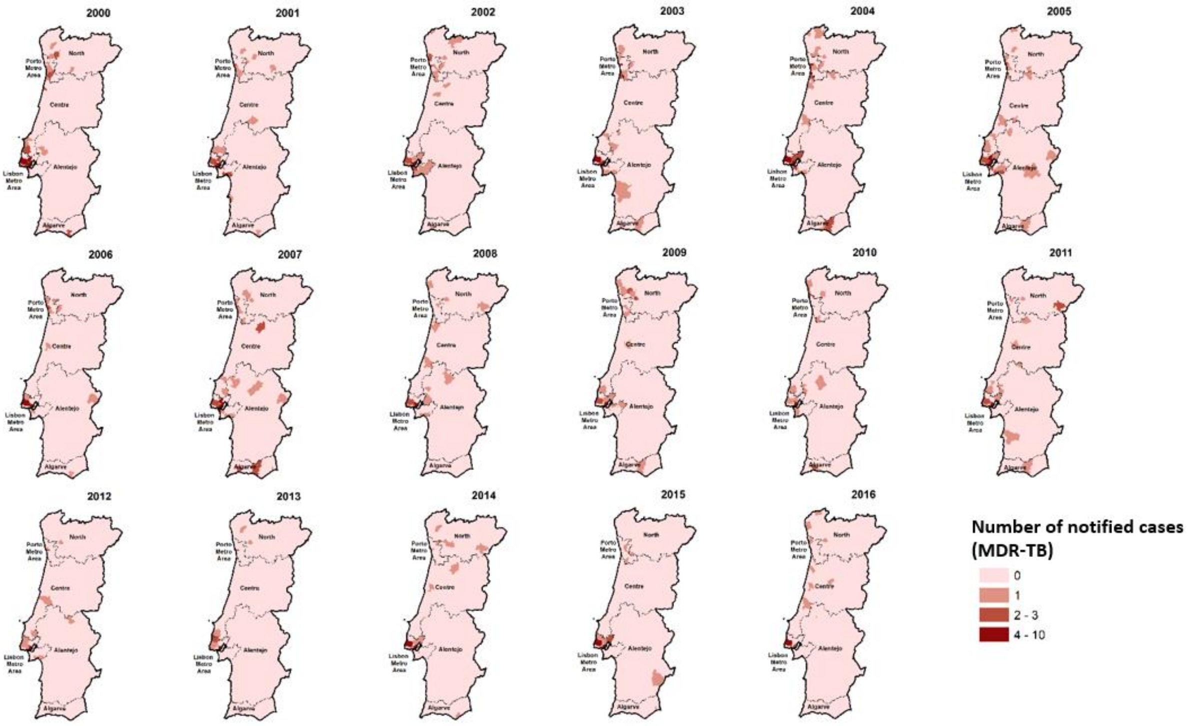

Supplementary Figure S2. Maps depicting a time series of the number of MDR-TB cases by municipality year by year, Continental Portugal, 2000-2016. TB=tuberculosis; MDR-TB= multidrug- resistant tuberculosis; the colour code displays the number of notified cases, from 0 to 10 cases.
